# The General Symptom Questionnaire-30 (GSQ-30): A Brief Measure of Multi-System Symptom Burden in Lyme Disease

**DOI:** 10.3389/fmed.2019.00283

**Published:** 2019-12-06

**Authors:** Brian A. Fallon, Nevena Zubcevik, Clair Bennett, Shreya Doshi, Alison W. Rebman, Ronit Kishon, James R. Moeller, Nadlyne R. Octavien, John N. Aucott

**Affiliations:** ^1^Department of Psychiatry, Lyme and Tick-Borne Diseases Research Center, Columbia University Irving Medical Center, New York, NY, United States; ^2^Department of Psychiatry, New York State Psychiatric Institute, New York, NY, United States; ^3^Department of Physical Medicine and Rehabilitation, Dean Center for Tick borne Illness, Harvard Medical School, Spaulding Rehabilitation Hospital, Boston, MA, United States; ^4^Department of Physical Medicine and Rehabilitation, Massachusetts General Hospital, Boston, MA, United States; ^5^Division of Rheumatology, Department of Medicine, Lyme Disease Research Center, Johns Hopkins School of Medicine, Baltimore, MD, United States

**Keywords:** Lyme disease, GSQ-30, PTLDS, multi-system illness, symptom burden

## Abstract

**Introduction:** The multi-system symptoms accompanying acute and post-treatment Lyme disease syndrome pose a challenge for time-limited assessment. The General Symptom Questionnaire (GSQ-30) was developed to fill the need for a brief patient-reported measure of multi-system symptom burden. In this study we assess the psychometric properties and sensitivity to change of the GSQ-30.

**Materials and Methods:** 342 adult participants comprised 4 diagnostic groups: Lyme disease (post-treatment Lyme disease syndrome, *n* = 124; erythema migrans, *n* = 94); depression, *n* = 36; traumatic brain injury, *n* = 51; healthy, *n* = 37. Participants were recruited from clinical research facilities in Massachusetts, Maryland, and New York. Validation measures for the GSQ-30 included the Patient Health Questionnaire-4 for depression and anxiety, visual analog scales for fatigue and pain, the Sheehan Disability Scale for functional impairment, and one global health question. To assess sensitivity to change, 53 patients with erythema migrans completed the GSQ-30 before treatment and 6 months after 3 weeks of treatment with doxycycline.

**Results:** The GSQ-30 demonstrated excellent internal consistency (Cronbach α = 0.95). The factor structure reflects four core domains: pain/fatigue, neuropsychiatric, neurologic, and viral-like symptoms. Symptom burden was significantly associated with depression (*r*_*s*_ = 0.60), anxiety (*r*_*s*_ = 0.55), pain (*r*_*s*_ = 0.75), fatigue (*r*_*s*_ = 0.77), functional impairment (*r*_*s*_ = 0.79), and general health (*r*_*s*_ = −0.58). The GSQ-30 detected significant change in symptom burden before and after antibiotic therapy; this change correlated with change in functional impairment. The GSQ-30 total score significantly differed for erythema migrans vs. three other groups (post-treatment Lyme disease syndrome, depression, healthy controls). The GSQ-30 total scores for traumatic brain injury and depression were not significantly different from post-treatment Lyme disease syndrome.

**Conclusions and Relevance:** The GSQ-30 is a valid and reliable instrument to assess symptom burden among patients with acute and post-treatment Lyme disease syndrome and is sensitive in the detection of change after treatment among patients with erythema migrans. The GSQ-30 should prove useful in clinical and research settings to assess multi-system symptom burden and to monitor change over time. The GSQ-30 may also prove useful in future precision medicine studies as a clinical measure to correlate with disease-relevant biomarkers.

## Introduction

Lyme disease is a serious and debilitating global illness, with estimated rates exceeding 400,000 new cases annually in the United States alone (1). While most patients recover fully after early detection and treatment, ~10% have symptoms that last 6 months or longer associated with functional impairment (“Post-treatment Lyme Disease Syndrome” or PTLDS) (2–4). Patients with persistent symptoms pose a challenge to clinicians as the symptoms vary across multiple medical domains, including the rheumatologic, neurologic, infectious, cardiac, psychiatric, and neurocognitive. This diversity of symptoms can make it difficult to assess treatment progress. Given the clinician's limited time with each patient, a brief self-report screening instrument covering multiple symptom domains would allow a rapid quantification of symptom burden, facilitate monitoring of change over time, and highlight other disease-relevant symptoms that require attention.

There are many somatic symptom scales that include symptoms commonly reported in the primary care setting (5–7). To our knowledge, there is only one self-report instrument specifically developed to address symptoms common to patients with Lyme disease. This instrument—the Horowitz Multiple Systemic Infectious Disease Syndrome Questionnaire—is a measure designed for the primary purpose of diagnosis of Lyme disease and other tick-borne disorders (8).

We designed the General Symptom Questionnaire (GSQ-30) to fill the need for a brief self-report instrument that assesses symptom burden and response to treatment among patients with multi-system disease. This instrument would be valuable in clinical trials and provide a quantitative clinical index for assessing the clinical relevance of biomarkers. While the GSQ-30 may be useful for monitoring a variety of multi-system medical conditions, it was designed specifically for patients with Lyme disease. In conducting this validation study, we hypothesized that the GSQ-30 would have good psychometric properties, be sensitive to detecting change after antibiotic treatment, and demonstrate clinically relevant profile differences between early and post-treatment Lyme disease symptoms and between Lyme disease and health. As a secondary goal, we examined whether the profile of PTLDS would differ from two similarly disabling conditions with multi-system symptoms- depression and traumatic brain injury (TBI).

## Materials and Methods

### Participants

Three hundred and forty-two participants, recruited across multiple sites, included 94 with early Lyme Disease who had a health-care provider diagnosed erythema migrans (EM) rash (*n* = 12 from the Lyme Center at Columbia University; *n* = 82 from the Lyme Center at Johns Hopkins University), 124 with IDSA case-defined PTLDS (*n* = 30 from Columbia; *n* = 94 from Johns Hopkins), 36 with depression from the New York State Psychiatric Institute (NYSPI), 51 with TBI from the outpatient brain injury clinic at Harvard's Spaulding Rehabilitation Hospital, and 37 healthy control participants (*n* = 14 from Columbia; *n* = 23 from Johns Hopkins).

The patients with EM had a rash with or without disseminated symptoms at study entry. The PTLDS patients met the IDSA case-definition which requires persistent symptoms that emerged during the first 6 months after antibiotic therapy for well-documented Lyme disease (4). The depressed participants had to score 14 or higher on the BDI-II indicating at least mild depression (*M* = 30.11, *SD* = 9.29). The TBI participants had to have a Glasgow Coma Scale score that fell in the mild (14–15) to moderate (9–13) range at least 18 months post-injury. Neither the depressed patients nor the TBI patients had a known history of Lyme disease. The healthy control participants were seronegative for *Borrelia burgdorferi* antibodies and free of symptoms associated with Lyme disease, medically healthy (Columbia site) or medically stable (Johns Hopkins site), had no history of major medical illness or severe viral-like symptoms in the last 6 months, and had no prior diagnosis or treatment for a tick-borne illness.

### Measures

**Figure 1:**
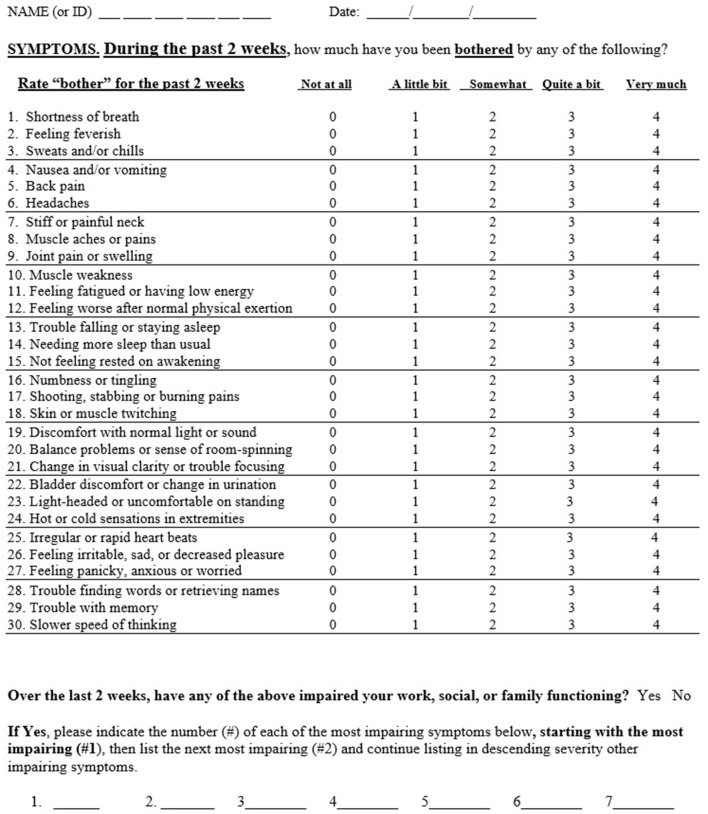
The general symptom questionnaire (GSQ-30).

The GSQ-30 is a 30 item questionnaire which assesses symptom burden over a 2 week time period (see Figure 1). Modeled after measures of somatic symptom burden in primary care, the PHQ-15 (5) and the SSS-8 (6), the GSQ-30 asks: “how much have you been bothered by any of the following?” with five options: “not at all,” “a little bit,” “somewhat,” “quite a bit,” and “very much” (scored 0–4); total score ranges from 0 to 120. The 2 week timeframe was selected to be shorter than the 1 month interval used for the PHQ-15 to minimize recall bias, and longer than the 1 week interval used for the SSS-8 to account for the waxing and waning nature of Lyme disease symptoms. The items selected for the GSQ-30 reflect somatic and neuropsychiatric symptoms commonly reported by patients with Lyme disease as noted in the literature (9–11) and from the authors' clinical research experience (BAF, NZ, JNA).An additional question (not included in the scoring) asks whether any of the above 30 items have impaired work, social or family functioning; the rater then lists the most impairing items in rank order of severity (up to seven items), thereby highlighting symptoms of most concern to the individual.

The Patient Health Questionnaire-4 (PHQ-4) (12) is an ultra-brief four item instrument with good psychometric properties developed to assess anxiety (items 1–2) and depression (items 3–4).

The Sheehan Disability Scale (SDS) (13), a valid and reliable measure of disability (14, 15), is a brief self-report measure designed to assess functional impairment across three domains of work/school, social and family life. The measure was adapted to span functioning “over the past 2 weeks.” The summed score for the three domains provides a measure of global functional impairment.

Visual analog scales (VAS) assessed pain and fatigue over the prior 2 weeks with scores ranging from 0 (“No___”) to 10 (“Most Severe___”). Visual analog scales have been shown to be valid and reliable in the assessment of pain (16), and are used in studies of fatigue (17, 18).

A single item, rated on a 5-point scale, was used to assess self-reported general health (“excellent,” “very good,” “good,” “fair,” or “poor”). This identical item is used in the SF-36 and the CDC HRQOL-4 Module (19, 20). Single item health questions are widely used in population-based research with demonstrated validity and reliability (21).

### Procedures

The GSQ-30 was included in ongoing IRB-approved research protocols at NYSPI and Johns Hopkins University during which all participants provided written informed consent. The Spaulding Rehabilitation Hospital/Partners Healthcare IRB authorized the retrospective collection of de-identified clinical data. The NYSPI/Columbia site served as the data coordinating center. All participants completed baseline self-report and demographic questionnaires. A subset of the individuals with EM from the Johns Hopkins site (*n* = 53) completed questionnaires again 6 months after treatment as part of a larger longitudinal cohort study. The pre-treatment and 6 month timepoints were used to assess change.

### Statistical Analysis

Internal consistency was assessed using Cronbach's alpha for the total GSQ-30 scale.

Construct validity was examined in three stages. (1) Bivariate correlations were examined between the GSQ-30, PHQ anxiety and depression totals, visual analog scales for pain and fatigue, the SDS total, and the general health item. Due to violations of normality, Spearman rank correlations were conducted with bootstrapped confidence intervals. (2) Sequential multivariable regression was employed, with functional impairment as the outcome variable, GSQ-30 as the predictor, and anxiety and depression scores entered at step 1 as covariates, to determine if symptom severity on the GSQ-30 improved prediction of functional impairment beyond the effects of anxiety/depression. Since regression diagnostics indicated some evidence of heteroscedasticity, a bootstrapped regression model was conducted. (3) Welch one-way tests with Holm correction for multiple comparisons were conducted to examine whether the GSQ-30 total could be used to distinguish between: (a) health status group based on the general health assessment; and (b) PTLDS, EM, depression, TBI, and healthy controls.

Factorial validity was examined with all participants except for healthy controls (*n* = 305) using principal components analysis (PCA) with an oblique “Promax” rotation to identify the number of components and determine the factor structure. PCA was conducted using polychoric correlations due to the ordinal nature of the data (22–24). The Kaiser-Meyer-Olkin measure of sampling adequacy was used to determine adequacy of sample size, and Bartlett's test of sphericity was used to assess suitability of the data for PCA. The number of components was determined by examining the number of eigenvalues >1, scree plot, parallel analysis (25) and significant factor loadings.

Sensitivity to change of the GSQ-30 was assessed in the subsample of patients with EM followed over time (*n* = 53). Treatment response at 6 months was categorized into three groups based on the presence of symptoms and/or functional impact, as previously described (2): “PTLDS” (i.e., symptoms with functional impairment), “Symptoms only” (i.e., symptoms without impairment), and “Returned to health.”

GSQ-30 scores before treatment were compared to scores 6 months later using a paired samples *t*-test. Percentage change in score from baseline to 6 months was calculated for 50 of the 53 participants from the EM subsample (three were not included due to baseline scores of 0). Percent change was again calculated separately for each outcome group. Given the non-normality of the data, both mean and median percent changes are reported.

In addition, a cross-sectional comparison was conducted among the outcome groups at 6 months using a Welch one-way test. *Post-hoc* tests with Holm correction for multiple comparisons were conducted to compare groups. A mixed ANOVA was also conducted to explore the interaction of time (i.e., pre- and post-treatment visits) and outcome group. Finally, the association between change in GSQ-30 scores and change in functional impairment scores from baseline to the 6 month follow-up was examined using Kendall's tau-b correlation of difference scores.

**Figure 2:**
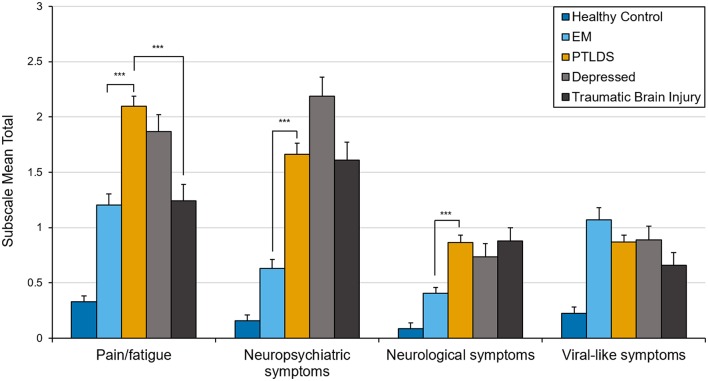
Mean total score on factor-derived subscales by clinical group. Error bars represent standard errors. ***p < 0.001.

In an exploratory analysis to determine whether the clinical profile of PTLDS differs from other clinically ill groups, subscale scores representing the mean of items within each of the 4 clusters identified in the PCA were compared and Welch one-way tests with Games-Howell *post-hoc* pair-wise comparisons were conducted. Group means in Figure 2 include the healthy sample group to aid in interpretation of clinical data. Paired samples *t*-tests were also conducted to examine change over time in the EM subsample using the newly derived subscales.

Missing data was present on key variables at a rate of <5% and imputed with a sequential hot-deck technique (26). All reported *p*-values were 2-sided, with *p* < 0.05 considered statistically significant. Statistical analyses were performed using R version 3.5.1 (27).

## Results

Demographics and clinical characteristics of the total sample and individual groups are presented in Table 1.

**Table 1:** Demographic and clinical characteristics of all groups.

	**Depressed** **(*n* = 36)**	**EM** **(*n* = 94)**	**Healthy control** **(*n* = 37)**	**PTLDS** **(*n* = 124)**	**Traumatic brain injury** **(*n* = 51)**	**Total** **(*n* = 342)**
Age, mean (SD), y	36.78 (11.42)	50.67 (14.93)	44.05 (16.18)	44.80 (15.37)	46.63 (16.97)	45.76 (15.66)
Sex (%)						
Female	23 (63.9)	48 (51.1)	25 (67.6)	54 (43.5)	24 (47.1)	174 (50.9)
Male	12 (33.3)	46 (48.9)	12 (32.4)	70 (56.5)	27 (52.9)	167 (48.8)
Other	1 (2.8)	0 (0.0)	0 (0.0)	0 (0.0)	0 (0.0)	1 (0.3)
Ethnicity (%)						
Black (non-Hispanic)	1 (2.8)	1 (1.1)	3 (8.1)	3 (2.4)	3 (5.9)	11 (3.2)
Hispanic	4 (11.1)	0 (0.0)	6 (16.2)	4 (3.2)	3 (5.9)	17 (5.0)
Other	7 (19.4)	2 (2.1)	7 (18.9)	5 (4.0)	3 (5.9)	24 (7.0)
White (non-Hispanic)	24 (66.7)	91 (96.8)	20 (54.1)	112 (90.3)	38 (74.5)	285 (83.3)
Not specified	0 (0.0)	0 (0.0)	1 (2.7)	0 (0.0)	4 (7.8)	5 (1.5)
Education (%)						
12 years or less	4 (11.1)	7 (7.4)	6 (16.2)	21 (16.9)	7 (13.7)	38 (11.1)
13 to 16 years	20 (55.6)	37 (39.4)	15 (40.5)	57 (46.0)	9 (17.6)	129 (37.7)
16 years or more	12 (33.3)	50 (53.2)	15 (40.5)	45 (36.3)	9 (17.6)	122 (35.7)
Not specified	0 (0.0)	0 (0.0)	1 (2.7)	1 (0.8)	26 (51.0)	53 (15.5)
Employment (%)^a^						
Disabled	–	1 (1.1)	0 (0.0)	4 (3.2)	51 (100.0)	56 (16.4)
F/T or P/T employed	–	67 (71.3)	22 (59.5)	77 (62.1)	0 (0.0)	166 (48.5)
F/T or P/T student	–	2 (2.1)	4 (10.8)	11 (8.9)	0 (0.0)	17 (5.0)
Homemaker	–	5 (5.3)	2 (5.4)	9 (7.3)	0 (0.0)	16 (4.7)
Other	–	2 (2.1)	0 (0.0)	5 (4.0)	0 (0.0)	7 (2.0)
Retired	–	4 (4.3)	3 (8.1)	5 (4.0)	0 (0.0)	12 (3.5)
Unemployed	–	13 (13.8)	5 (13.5)	13 (10.5)	0 (0.0)	31 (9.1)
Not specified	–	0 (0.0)	1 (2.7)	0 (0.0)	0 (0.0)	37 (10.8)
General health, mean (SD)	2.89 (0.89)	3.85 (0.89)	3.95 (0.66)	2.81 (1.02)	3.12 (1.05)	3.27 (1.06)
Pain, mean (SD)	4.19 (2.45)	2.98 (2.81)	0.76 (0.98)	4.58 (2.25)	3.20 (3.03)	3.48 (2.72)
Fatigue, mean (SD)	6.39 (2.41)	3.91 (3.12)	1.35 (1.36)	5.60 (2.45)	4.25 (3.08)	4.56 (3.00)
Anxiety, mean (SD)	3.17 (2.06)	0.83 (1.41)	0.57 (1.14)	1.66 (1.84)	1.84 (2.06)	1.50 (1.87)
Depression, mean (SD)	3.97 (1.76)	0.70 (1.12)	0.22 (0.85)	1.47 (1.53)	1.92 (2.11)	1.45 (1.81)
Functional impairment, mean (SD)	16.69 (8.40)	8.87 (10.09)	1.05 (2.78)	14.19 (8.93)	10.94 (10.34)	11.09 (9.97)

a *Employment was categorized differently for the depression group as: employed/not employed (75%, n = 27; 25%, n = 9)*.

### Internal Consistency

Cronbach's alpha for the GSQ-30 total score was excellent, *r* = 0.95. Internal consistency was high for all groups when assessed separately including the healthy controls, EM, PTLDS, TBI, and depression patients; *r* = 0.86, 0.94, 0.93, 0.96, and 0.93, respectively.

### Construct Validity

GSQ-30 total scores including all groups were significantly correlated with depression (*r*_*s*_ = 0.60, 95% CI[0.53, 0.66]), anxiety (*r*_*s*_ = 0.55, 95% CI[0.48, 0.62]), pain (*r*_*s*_ = 0.75, 95% CI[0.68, 0.80]), fatigue (*r*_*s*_ = 0.77, 95% CI[0.72, 0.80]), functional impairment (*r*_*s*_ = 0.79, 95% CI[0.75, 0.83]), and general health (*r*_*s*_ = −0.58, 95% CI[−0.65, −0.50]), all at *p* < 0.001.

The sequential regression analyses demonstrated that depression and anxiety significantly predicted functional impairment (*R*^2^ = 0.377, 95% CI[0.30, 0.46], *p* < 0.001). Addition of the GSQ-30 total score resulted in a significant Δ*R*^2^ of 0.244 (95% CI[0.17, 0.32], *p* < 0.001), indicating that the GSQ-30 total predicted functional impairment over and above symptoms of anxiety/depression. Further, when the GSQ-30 was added as the first predictor, it accounted for 57% (95% CI[0.50, 0.63]) of the variance in functional impairment with depression/anxiety contributing only an additional 5% (95% CI[0.02, 0.09]) when added later.

The Welch's one-way test with *post-hoc* comparisons indicated a significant difference between all health status groups on the GSQ-30 [*F*_(4, 95.28)_ = 74.96, *p* < 0.001]. GSQ-30 mean scores increased stepwise with each categorical decrease in reported general health (“excellent”(*M* = 13.49, *SD* = 14.2); “very good”(*M* = 20.73, *SD* = 19.7); “good”(*M* = 34.67, *SD* = 20.95); “fair”(*M* = 47.82, *SD* = 20.81); “poor”(*M* = 71.22, *SD* = 13.05); *p*-values ranged between <0.001 and 0.01.

There was a statistically significant difference between EM, PTLDS, depression, TBI, and healthy controls as determined by Welch's one-way test [*F*_(4, 128.69)_ = 77.79, *p* < 0.001]. *Post-hoc* tests indicated significant differences (all *p*-values < 0.001) on the GSQ-30 total score between the healthy control group (*M* = 6, *SD* = 7.37), and all other groups: EM (*M* = 24.15, *SD* = 20.11), PTLDS (*M* = 42.38, *SD* = 22.14), depressed (*M* = 42.28, *SD* = 21.05) and TBI (*M* = 32.82, *SD* = 26.79). The GSQ-30 total score for the EM group was also significantly different from PTLDS and depression. No other comparisons were significant.

### Factorial Validity

PCA was conducted with data from all participants except healthy controls. The Kaiser-Meyer-Olkin measure verified the sampling adequacy for the analysis, KMO = 0.86, indicating a “meritous” degree of common variance (28, 29) and Bartlett's test of sphericity was significant (*p* < 0.001), indicating sufficiently large correlations between items for PCA. Initial examination of eigenvalues revealed five components with eigenvalues >1. The parallel test and the scree plot suggested a more conservative 4-component solution. Given convergence of the scree plot and the parallel test, and the tendency for Kaiser's criterion to overestimate the true number of components (30), a 4-component solution was examined.

Items with component loadings ≥0.40 were retained. After the first rotation, two items (“trouble falling or staying asleep” and “needing more sleep than usual”) were removed due to loadings <0.40. The final solution accounted for 65.11% of variance. The item clusters suggested that component 1 represented neuropsychiatric problems and explained 17.11% of the variance, component 2 represented neurological symptoms and explained 18.93%, component 3 represented pain and fatigue symptoms and explained 16.93%, and component 4 represented viral-like symptoms and explained 12.14%. See Table 2 for component loadings. Cronbach's alpha for the GSQ-30 total score did not change substantially as a result of removing the two items, *r* = 0.94. The PCA was also run using only the Lyme participants (EM and PTLDS) as a supplementary analysis to examine factorial invariance. A similar structure emerged except: (a) the two sleep items were retained within the pain/fatigue component; and (b) the item ‘light-headed or uncomfortable on standing’ loaded on the viral-like symptoms component. See Table 3 for component loadings of the PCA for the Lyme sample. Due to the presence of a Heywood case, the solution reported used an “Oblimin” rotation rather than “Promax.”

**Table 2:** Summary of factor loadings for all clinical cases (*N* = 305).

	**NeuroΨ symptoms**	**Neurological symptoms**	**Pain/fatigue symptoms**	**Viral-like symptoms**
Slower speed of thinking	0.89			
Trouble with memory	0.88			
Trouble finding words or retrieving names	0.88			
Feeling panicky, anxious or worried	0.82			
Feeling irritable, sad, or decreased pleasure	0.81			
Hot or cold sensations in extremities		0.70		
Balance problems or sense of room-spinning		0.69		
Bladder discomfort or change in urination		0.65		
Numbness or tingling		0.64		
Skin or muscle twitching		0.59		
Change in visual clarity or trouble focusing		0.56		
Light-headed or uncomfortable on standing		0.53		
Irregular or rapid heart beats		0.53		
Discomfort with normal light or sound		0.52		
Shooting, stabbing or burning pains		0.50	0.43	
Shortness of breath		0.45		
Muscle aches or pains			0.92	
Joint pain or swelling			0.87	
Muscle weakness			0.75	
Back pain			0.66	
Feeling worse after normal physical exertion			0.58	
Stiff or painful neck			0.58	
Feeling fatigued or having low energy	0.40		0.51	
Not feeling rested on awakening	0.44		0.46	
Feeling feverish				0.99
Sweats and/or chills				0.85
Headaches				0.58
Nausea and/or vomiting				0.55

*Loadings of <0.40 are not displayed*.

**Table 3:** Summary of factor loadings for EM and PTLDS (*N* = 218).

	**Pain/fatigue symptoms**	**Neuropsychiatric symptoms**	**Neurological symptoms**	**Viral-like symptoms**
Muscle aches or pains	0.92			
Joint pain or swelling	0.76			
Muscle weakness	0.76			
Stiff or painful neck	0.65			
Back pain	0.65			
Feeling worse after normal physical exertion	0.6			
Not feeling rested on awakening	0.56			
Feeling fatigued or having low energy	0.54	0.43		
Needing more sleep than usual	0.49			
Trouble falling or staying asleep	0.48			
Slower speed of thinking		0.85		
Trouble with memory		0.84		
Trouble finding words or retrieving names		0.84		
Feeling panicky, anxious or worried		0.81		
Feeling irritable, sad, or decreased pleasure		0.74		
Hot or cold sensations in extremities			0.64	
Skin or muscle twitching			0.63	
Numbness or tingling			0.58	
Bladder discomfort or change in urination			0.55	
Balance problems or sense of room-spinning			0.54	
Change in visual clarity or trouble focusing			0.51	
Shortness of breath			0.5	
Irregular or rapid heart beats			0.5	
Shooting, stabbing or burning pains			0.45	
Discomfort with normal light or sound			0.43	
Feeling feverish				0.91
Sweats and/or chills				0.75
Headaches				0.54
Nausea and/or vomiting				0.5
Light-headed or uncomfortable on standing				0.43

*Loadings of <0.40 are not displayed*.

### Sensitivity to Change

Among the 53 patients with EM who had baseline and 6 month ratings, GSQ-30 total scores decreased significantly from baseline (*M* = 21.93, *SD* = 20.60) to 6 months post-treatment (*M* = 13.06, *SD* = 15.56; *p* < 0.01), with median and mean percent change in symptoms over the 6 month period of 51.87% (IQR = 1.44–89.29%) and 16.69%(SD = 102.38), respectively. The range (−300% to 100%) included participants with deterioration and improvement of symptoms over time. The percentage change over time for the three outcome groups differed as expected: Returned to health (*Md* = +80.68%*/M* = +50.55%), Symptoms only (*Md* = +24.59/*M* = −38.55%), PTLDS (*Md* = −16.56%/*M* = −38.56%).

**Figure 3:**
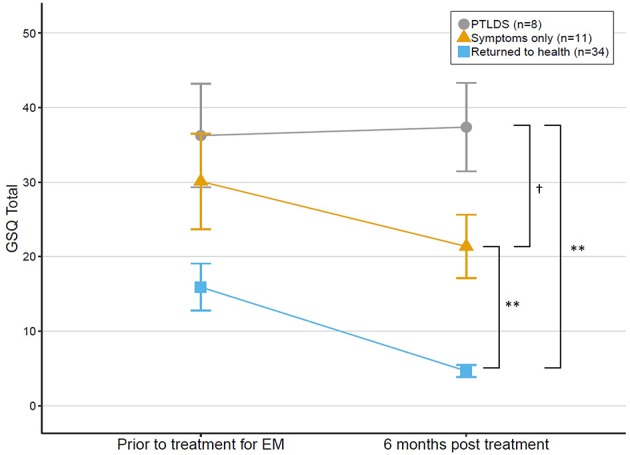
Change in GSQ-30 scores among patients treated for EM at baseline and reassessed 6 months later, grouped by outcome status at 6 months. Error bars represent standard errors. ^†^*p* = 0.05, **p < 0.01.

While most of the 53 patients with EM recovered fully (*n* = 34), others had symptoms without functional impairment (*n* = 11) and a smaller group had symptoms with functional impairment (PTLDS) (*n* = 8). There was a statistically significant difference in GSQ-total score between outcome groups at 6 months [*F*_(2, 11.52)_ = 20.68, *p* < 0.001]. *Post-hoc* tests revealed that all pairwise outcome group comparisons were significantly different; see Figure 3. Return to health (*M* = 4.65, *SD* = 4.68) was significantly different from both Symptoms only (*p* = 0.006) and PTLDS (*p* = 0.003). PTLDS (*M* = 37.38, *SD* = 16.77) was different from Symptoms only (*M* = 21.36, *SD* = 14.14) (*p* = 0.046).

Results from the mixed ANOVA indicated that there was a significant main effect of outcome group on the GSQ total score [*F*_(2, 50)_ = 20.36, *p* < 0.001, generalized η^2^ = 0.32], and time [*F*_(1, 50)_ = 10.81, *p* < 0.01, generalized η^2^ = 0.08], but that there was no interaction between group and time [*F*_(2, 50)_ = 1.29, *p* = 0.28, generalized η^2^ = 0.02]; this indicates that change in GSQ total score from baseline over time was not significantly different between outcome groups. Change in GSQ-30 total score including all 53 EM patients from baseline to 6 month follow-up was significantly correlated with change in functional impairment (*r*_τ_ = 0.61, *p* < 0.001).

### Exploratory Analyses With Factor-Derived Subscales

Welch's one-way tests indicated significant differences between clinical groups across three of four subscales; pain and fatigue [*F*_(3, 114.86)_ = 17.04, *p* < 0.001], neuropsychiatric symptoms [*F*_(3, 110.45)_ = 36.19, *p* < 0.001], and neurological symptoms [*F*_(3, 108.29)_ = 11.96, *p* < 0.001]. Pairwise *post-hoc* comparisons (Figure 2) revealed significant differences between PTLDS and both EM and TBI on the pain and fatigue subscale; and between PTLDS and EM on both the neurologic and the neuropsychiatric subscales. All other comparisons were not significant.

Using the factor-derived subscales, paired samples *t*-tests were conducted to examine change over time in the EM group for participants with available 6 month follow-up data (*n* = 53). Results demonstrated a significant reduction in pain/fatigue and viral-like symptoms for patients with a documented EM rash (see Table 4).

**Table 4:** *t*-tests with factor-derived subscales and total GSQ scores for antibiotic-treated EM cases.

**Scale**	**Baseline** ***M* (SD)**	**6-months** ***M* (SD)**	***t***	***P* value**	**Cohen's *d***
Pain/Fatigue	1.09 (0.96)	0.70 (0.79)	2.92	<0.01	0.45
Neuropsychiatric	0.54 (0.75)	0.51 (0.68)	0.34	0.74	0.05
Neurological symptoms	0.37 (0.53)	0.25 (0.45)	1.95	0.06	0.25
Viral-like symptoms	0.96 (0.98)	0.26 (0.43)	4.94	<0.001	0.91
Total GSQ (summed)	21.93 (20.60)	13.06 (15.56)	3.27	<0.001	0.49

## Discussion

The GSQ-30 is a psychometrically sound measure of symptom burden among patients with multi-system illness. It has the advantages of brevity, ease of administration and scoring, and sensitivity to change after treatment. Using a multi-site cohort of 342 participants, the GSQ-30 demonstrated excellent internal consistency among items. Not surprisingly, the GSQ-30 was significantly associated with other construct-related measures such as brief scales of depression, anxiety, fatigue, pain, and general health. That the GSQ-30 is not simply another way of assessing anxiety or depression was supported by the regression analysis which indicated that the GSQ-30 accounted for an additional 25% of variance in the functional impairment score beyond that contributed by anxiety and depression.

Notably, the GSQ-30 total score correlated strongly with functional impairment. In addition, reduction in the GSQ-30 over time corresponded with improvement in functional status. These findings support the conclusion that the GSQ-30 detects symptom impact (i.e., burden) and not just presence.

The factor analysis led to the identification of four core domains: viral-like, pain/fatigue, neurologic, and neuropsychiatric symptoms. These are common symptom clusters reported by patients impacted by Lyme disease. The domain profile of PTLDS differed from that of EM, with the former having a significantly greater burden of pain/fatigue, neuropsychiatric, and neurologic symptoms. These results, as well as the finding that the GSQ-30 total score for PTLDS was nearly 2x higher than for EM, support the clinical impression that patients with PTLDS have a much greater symptom burden than those with early Lyme disease.

The analysis of change among patients with EM after antibiotic treatment identified significant improvement over time in both total score as well as in subscales of pain/fatigue and viral-like symptoms. That significant improvement was not seen in the subscales of neurologic and neuropsychiatric symptoms raises several questions. Are these domains reflective of symptoms triggered by infection but not due to persistent infection? Is a different antibiotic or another mode of treatment (e.g., anti-inflammatory or neuromodulatory interventions) needed to reduce symptoms in these domains? Can the subscale scores on the GSQ-30 be used to guide treatment planning? These clinically important questions can be addressed in future research.

This study demonstrated that the research algorithms used to categorize patients' treatment response as “Returned to health,” “Symptoms only,” or “PTLDS” correspond with scores on the GSQ-30. The “Returned to health” group had significantly lower GSQ-30 total scores (mean 6 and 4.65, respectively) compared to the Symptoms only group (mean 21.36) which in turn had significantly lower scores than the PTLDS group (mean 37.38). Poor outcome at 6 months may be due to many causes, including persistent infection, post-infectious processes or re-infection. Regular administration of the GSQ-30 may improve outcome by highlighting for the clinician the symptoms of greatest burden to the patient which may need a different treatment approach. Strikingly, the GSQ-30 total score for the EM patients who developed PTLDS on average was high at the first assessment and remained high at the 6 month assessment after treatment, while the recovered group had markedly lower scores at baseline which declined with treatment (Figure 3). This raises the possibility that the magnitude of the GSQ-30 at initial evaluation may identify a subgroup of EM patients in need of treatment augmentation to increase the likelihood of improved long-term outcome. Whether the GSQ-30 total score corresponds with particular biomarkers, such as inflammatory cytokines, would be of great interest for future exploration. The GSQ-30 therefore appears to be a useful instrument to complement clinical judgment and ratings of symptom burden.

We examined how patients with PTLDS compared to those with depression and TBI on the GSQ-30 total and subscale scores. The lack of a significant difference in total scores may reflect the multi-system involvement in all three disorders. This highlights that the GSQ-30 is a measure of symptom burden and not a diagnostic instrument. The subscales however may reveal symptom profiles that differ between disorders, as in the contrast between PTLDS and TBI on the pain/fatigue subscale. The lack of difference between PTLDS and depression in both total and subscale scores highlights the striking overlap between these two disorders. Both are associated with disturbances of energy, sleep, cognition, pain, and mood and both may be mediated by common central nervous system immune mechanisms (31, 32).

The strengths of this study include the large sample size, the selection of items common to patients with early and post-treatment Lyme disease syndrome, the identification of subscales statistically that have clinical face validity, and the demonstration of significant change using prospectively collected data among patients with EM before and after standardized treatment. The primary limitation of this study is that we could not assess sensitivity to change of the GSQ-30 in the PTLDS group, as we did not have access to a prospectively treated group of PTLDS patients before and after treatment. PTLDS is a more heterogeneous condition than EM; this may impact the ability of the GSQ-30 to assess change over time. A second limitation is that although the healthy control group was required to be seronegative for *B. burgdorferi* antibodies, the TBI and depressed patients were not serologically tested; therefore, we cannot rule-out prior unrecognized infection with *B. burgdorferi* in some of the latter patients.

Future studies should examine the usefulness of the GSQ-30 in other infected cohorts (e.g., *Babesia microti, Borrelia miyamotoi*), the relationship of the GSQ-30 to specific biomarkers, and whether clinical outcome can be improved by using the GSQ-30 to guide treatment reassessment.

In conclusion, the GSQ-30 is a valid and reliable instrument to assess symptom burden among patients with acute and post-treatment Lyme disease syndrome and is sensitive in the detection of change after antibiotic treatment among individuals with EM.

## Data Availability Statement

Participant consent did not include seeking permission for data to be made publicly available.

## Ethics Statement

This study, involving human participants, was reviewed and approved by each institution's respective IRB (The New York State Psychiatric Institute, Johns Hopkins University School of Medicine, Partners Health Care). The patients/participants provided their written informed consent to participate in this study.

## Author Contributions

BA and JA had full access to all the data in the study and take responsibility for the integrity of the data and the accuracy of the data analysis. CB and JM conducted the statistical analyses. BF, SD, NZ, and JA take responsibility for the study's concept and design. BF, CB, SD, JA, and AR participated in the drafting of the manuscript. BF obtained funding. SD, AR, and NO provided administrative, technical, or material support. BF, JA, and NZ provided supervision. All authors contributed to the acquisition, analysis or interpretation of the data and all contributed to critical revision of the manuscript for important intellectual content.

### Conflict of Interest

The authors declare that the research was conducted in the absence of any commercial or financial relationships that could be construed as a potential conflict of interest.
